# Personal electric deterrents can reduce shark bites from the three species responsible for the most fatal interactions

**DOI:** 10.1038/s41598-024-66679-6

**Published:** 2024-07-15

**Authors:** Thomas M. Clarke, Adam Barnett, Richard Fitzpatrick, Laura A. Ryan, Nathan S. Hart, Arnault R. G. Gauthier, Tracey B. Scott-Holland, Charlie Huveneers

**Affiliations:** 1 Southern Shark Ecology Group, College of Science and Engineering, Flinders University, Adelaide, SA 5042 Australia; 2 Marine Data Technology Hub, James Cook University, Townsville, QLD Australia; 3 Biopixel Oceans Foundation, Cairns, QLD Australia; 4 School of Natural Sciences, Macquarie University, North Ryde, NSW 2109 Australia; 5 Centre Sécurité Requin, 25F Avenue Des Artisans, Zone Artisanale de La Pointe Des Châteaux, 97436 Saint Leu, Reunion Island France; 6 Department of Agriculture and Fisheries, Brisbane, QLD 4000 Australia

**Keywords:** Behavioural ecology, Feeding behaviour, Marine biology

## Abstract

The frequency of unprovoked shark bites is increasing worldwide, leading to a growing pressure for mitigation measures to reduce shark-bite risk while maintaining conservation objectives. Personal shark deterrents are a promising and non-lethal strategy that can protect ocean users, but few have been independently and scientifically tested. In Australia, bull (*Carcharhinus leucas*), tiger (*Galeocerdo cuvier*), and white sharks (*Carcharodon carcharias*) are responsible for the highest number of bites and fatalities. We tested the effects of two electric deterrents (Ocean Guardian’s Freedom+ Surf and Freedom7) on the behaviour of these three species. The surf product reduced the probability of bites by 54% across all three species. The diving product had a similar effect on tiger shark bites (69% reduction) but did not reduce the frequency of bites from white sharks (1% increase), likely because the electrodes were placed further away from the bait. Electric deterrents also increased the time for bites to occur, and frequency of reactions and passes for all species tested. Our findings reveal that both Freedom+ Surf and Freedom7 electric deterrents affect shark behaviour and can reduce shark-bite risk for water users, but neither product eliminated the risk of shark bites entirely. The increasing number of studies showing the ability of personal electric deterrents to reduce shark-bite risk highlights personal protection as an effective and important part of the toolbox of shark-bite mitigation measures.

## Introduction

Human-shark interactions have been steadily increasing over the past ~ 40 years^1–3^. Possible causes of the continued rise in global shark incidents remain a debated and contentious topic, and are often associated with human population growth in coastal areas and increases in water-based activities such as surfing and diving^2,4,5^. However, environmental and habitat variation, such as changing ocean temperature^4^, decreased water clarity^6,7^, and climate change^4^ may also contribute to the rising number of shark bites on humans^8^. Despite the overall risk to water users remaining low and infrequent, and most often resulting in only minor injuries^5,9^, increasing concern perpetuated among the general public and mass media has contributed to a need for protective measures to alleviate some of the public safety concerns^10–13^.

Shark-bite mitigation measures include localised and/or broad-scale culling programs, swimming enclosures, beach nets, drumlines, land- and aerial-based shark spotting, education (e.g., SharkSmart), and acoustic tracking^14,15^. These measures can improve the safety of water users^16,17^, but have either raised conservation or ethical concerns (i.e., lethal methods; Ref.^18^) or are not suitable across all water users. For example, enclosures are only suitable for bathers in areas protected from large swells and land or aerial shark spotting is not applicable to divers^16^. Exclusion barriers that incorporate magnets^19^ or electromagnets^20^ have been developed for exposed conditions, but they do not stop all sharks and are expensive to deploy and maintain across large areas. There remains a need for measures protecting surfers and divers, which represents a large proportion of shark-bite victims^9^. More recently, public support for traditional, lethal measures of shark-bite mitigation has declined as alternative, non-lethal methods, e.g., early-warning systems and Shark-Management-Alert-In-Real-Time (SMART) drumlines, have increasingly gain attention and traction^21–24^. For example, 65% of water users from New South Wales (Australia) slightly, moderately, or strongly agree with personal deterrents as a management option to mitigate shark risk^21–25^. Among these alternatives, a range of personal deterrents have been developed and are commercially available. These deterrents have been designed to deter sharks by disrupting one or more of their senses, e.g., vision, smell, taste, magnetoreception, or electroreception^26–28^. One such class of sensory-based deterrent are electric deterrents, which function by producing a strong, pulsed electrical field that is designed to overwhelm the highly sensitive electrosensory system of sharks, and are one of the few types of commercially-available devices that have been scientifically tested and shown to reduce the risk of shark bites^28–31^.

Electroreception in sharks occurs via specialised receptors, the ampullae of Lorenzini, and enables the detection of weak electrical potentials generated by living (and some inanimate) objects in the water. Sharks use this electrosensory system for predator avoidance^32,33^, to maintain orientation and position^34,35^ and to locate nearby prey^34,36–38^. The ability of elasmobranchs to detect electromagnetic fields is species-specific and influenced by morphology, habitat, and foraging strategies^37,39^, and can even vary within species^40,41^ or between freshwater and marine habitats within individuals (e.g., *C. leucas*, ^42^). Variations in the sensitivity of the electrosensory system in otherwise morphologically-similar species are attributed to differences in ampullae distribution^43–45^, ampullary canal length^46,47^, and the number of alveoli^47^. Given that the electrosensory system of sharks is so strongly linked to feeding behaviour, intra- or interspecific differences in the sensitivity and/or higher processing of electrosensory information might be expected to influence the effectiveness of electric fields, such as those emitted by electric shark deterrents, in deterring sharks^30,48^.

The shark species responsible for the most unprovoked bites and related fatalities are the bull *Carcharhinus leucas*, tiger *Galeocerdo cuvier*, and white shark *Carcharodon carcharias*^8,9^, which are thus the focus of most shark-bite mitigation strategies. All three species can inhabit coastal and pelagic habitats^49^, but differ in their neuroanatomy of sensory organs, including ampullae of Lorenzini. For example, bull sharks possess the highest number of electroreceptive pores (quantity ± standard deviation: 1,852 ± 59.8), followed by white (812 ± 134) and tiger sharks (798 ± 24; Ref.^39,49^). Rainfall and turbidity are also key drivers of the occurrence of bull sharks in near-shore areas^50^, with most bites on humans in Australia occurring in coastal turbid areas^9^, suggesting that bull sharks might use electroreception more than sight during foraging events. Electric deterrents may therefore be more effective at reducing frequency of bites from bull sharks than tiger or white sharks.

Of the commercially available personal electric deterrents, the products most extensively tested scientifically are the Ocean Guardian (previously called Shark Shield) diving and surfing products. The ability of Shark Shield/Ocean Guardian products to reduce the risk of shark bites have been tested on white sharks^28–30,51–53^, bull sharks^31^, and blacktip reef sharks (*Carcharhinus melanopterus*; Ref.^54^). Overall, these studies show that Shark Shield/Ocean Guardian products can reduce shark bites by ~ 60% across the species tested. However, electric deterrents could not reduce bycatch of sawfish (largetooth sawfish, *Pristis pristis*) in a trawl fishery^55^. Electric deterrents have, so far, not been tested on tiger sharks, despite this species being one of the three species responsible for the most unprovoked shark bites^9^. Here, we build on the previous testing by assessing the effectiveness of two commercially-available electric shark deterrents, i.e., Ocean Guardian Freedom+ Surf (surfing product) and Freedom7 (diving product), on tiger sharks and compare results to previous studies testing the effectiveness of these products on bull and white sharks. We hypothesise that both products will reduce the probability of a bite from bull, tiger, and white sharks, and increase the time for the bite to occur, number of passes, and number of reactions. Based on assumed differences in electrosensory sensitivity among species, we hypothesised that electric deterrents will be more effective on bull than tiger or white sharks. We also developed a novel method to characterise shark behaviour and assess behavioural changes in tiger sharks exposed to electric deterrents. With this study, personal electric shark deterrents have been tested on the three species most responsible for severe shark bites globally, providing insight into the ability to generalise the effectiveness of electric deterrents on coastal-pelagic species.

## Methods

**Figure 1:**
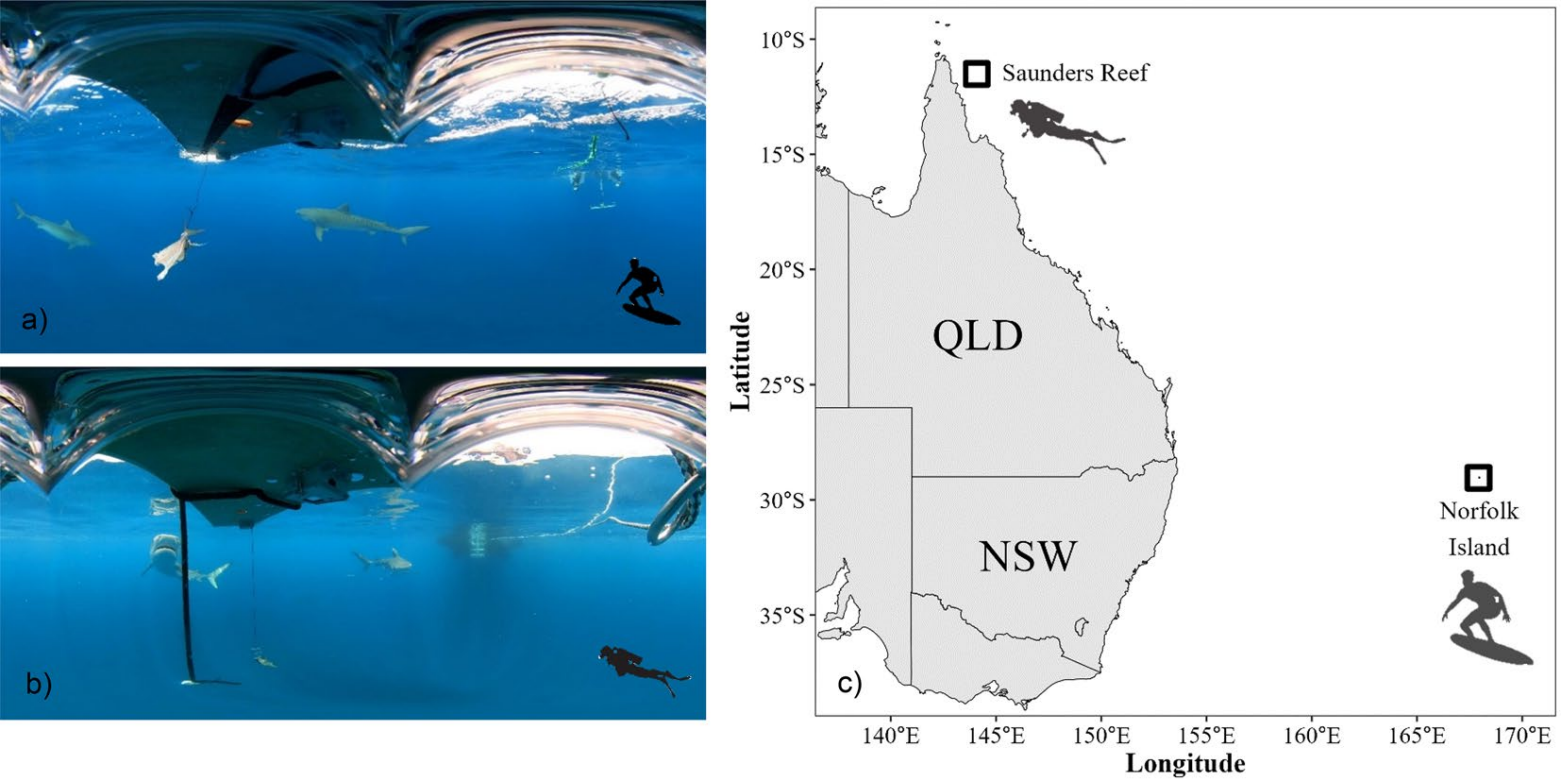
Sub-surface views taken from 360-degree cameras during tiger shark trials of trial set-ups for a) Freedom+ Surf, and b) Freedom7, and c) location of trials. Map was created in R (version 3.3.0) with RStudio (version 2023.06.2), using the ggplot2 package (version 3.4.3).

We tested the two Ocean Guardian products (ocean-guardian.com) on tiger sharks following the same protocol as used for bull^31^ and white sharks^28,29^ to facilitate comparison. We deployed a 1.2 × 0.4 m fibreglass-coated wooden board ~ 10 m away from the stern of the research vessel for 15 min or until the bait was taken or the board was bitten. Tiger sharks were attracted to the vessel by dispersing a berley mix of minced local fish (e.g., *Sardinops* spp.) and tethered baits behind the vessel. We commenced trials once a tiger shark was sighted at least twice within 3 min and showed consistent interest in the tethered bait. Trial baits (head or frame of a local fish, ~ 0.5 m length) was suspended ~ 0.5 m below the board and centred between the two electrodes, with the bait ~ 0.5 m away from both electrodes (Fig. 1). The position of the bait was designed to replicate the lower leg and foot of a surfer while sitting on a surfboard and waiting for waves (Freedom+ Surf, Fig. 1a) or that of a diver or snorkeler’s leg wearing the deterrent (Freedom7, Fig. 1b). For each trial, we deployed either an active deterrent (*Treatment*) or decoy (*Control*) using block randomisation. We recorded the interactions between tiger sharks and the board replica using a sub-surface 360-camera (Insta360 ONE X2, insta360.com). We repeated trials during which a shark did not approach the board with an intent to take the bait to ensure that the results were not biased by trials during which sharks did not attempt to consume the bait. Total length of tiger sharks interacting during trials were recorded based on visual estimates by experienced researchers^56^. We tested the Freedom+ Surf on tiger sharks off Headstone Bay in Norfolk Island, a small remote island in the Pacific Ocean, ~ 1,400 km east of Australia (29°02′48.3″S 167°55′07.9″E; Fig. 1b). Freedom7 tiger shark trials were undertaken off Saunders Reef, a remote tropical reef in the far north Great Barrier Reef, Australia (11°30′14.6″S 144°04′21.5″E, Fig. 1). Bull shark trials were undertaken in Noumea, New Caledonia^31^ but only the Freedom+ Surf was tested. White shark testing occurred at the Neptune Islands Group in South Australia, and were sourced from^28^ and^29^. All bull and white shark trials were recorded via GoPro Hero 3/4/7 models in underwater housings. All methods in this study were performed in accordance with guidelines and regulations approved by Flinders University research ethics. Testing of electric deterrents on tiger sharks was undertaken under Flinders University Animal Ethics Approval to test the efficacy of shark deterrents: project number BIOL4985-2 (tiger sharks), E3446 (bull sharks and white sharks). This study is reported in accordance with Animal Research: Reporting of In Vivo Experiments (ARRIVE) guidelines (arriveguidelines.org).

Tiger shark 360-degree videos were coded using Behavioural Observation Research Interactive Software (BORIS, version 7.12.2; Ref.^57^). To remain consistent with bull and white shark trials, we measured four response variables, (1) whether the board or bait was bitten (hereafter we refer to those as ‘bites’), (2) time for bites to occur, (3) number of passes, and (4) number of reactions (Table 1). We defined *passes* as the shark swimming towards the board (each time a shark veered away from the board and swam back towards it was classified as a new pass)^29^. We also developed a novel method to characterise tiger shark behaviour using 360-degree video footage. We created a tiger shark behaviour ethogram combining previous descriptions of pelagic shark behaviour^58–63^ and behaviours observed during previous deterrent testing^28,31^. We identified five tiger shark behaviours and recorded the duration (in seconds, *time spent*) of each behaviour for each shark and trial: (1) *approach*, period when shark swam towards the board within 2–3 body length with an intent to make contact; (2) *swim away*, period when shark swam away from the board following an approach, (3) *patrolling*, period when shark swam in a straight line, more than 2–3 body length from the board, with no apparent interest in the board or bait^58,62^, (4) *glide*, period of slow, horizontal swimming with no tail beat^63^; and (5) *out*, period where shark was no longer visible after first being sighted. We identified individual sharks using unique markings and colouration^64^. Sharks that could not be identified confidently were assigned as ‘unknown’.

**Table 1:** Summary of response variables tested on bull, tiger, and white sharks in response to electric shark deterrents. # = Number, = Time; Surf = Freedom+ Surf, F7 = Freedom7.

	# Bite		Bite		# Passes		# Reactions		Behaviour state	
	Surf	F7	Surf	F7	Surf	F7	Surf	F7	Surf	F7
Bull shark	×	✓	×	✓	×	✓	×	✓	×	×
Tiger shark	✓	✓	✓	✓	✓	✓	✓	✓	✓	✓
White shark	✓	✓	✓	✓	✓	✓	✓	✓	×	×

All analyses were performed in the R statistical environment (version 4.0.2). We tested the effects of each deterrent on all four response variables for bull, tiger, and white sharks (Table 1) using a combination of generalised linear effects models (GLMs; no random effects) and generalised linear mixed effects models (GLMMs; random effects) using the *glm* and *lmer* functions in the lme4 package (version 1.1.23; Ref.^65^), and Generalised Additive Mixed Models (GAMMs) when the relationship between the response variable and predictors was expected to be non-linear using the *gam* function in the mgcv package (version 1.8.33). Time spent in each behaviour state was tested for tiger sharks, but were not recorded in previous studies, and therefore species could not be included in the model testing for the effects of the deterrent on behaviours. Potential temporal effects were accounted for by including trial set (trial) as a fixed-integer effect, and nesting trial sets within corresponding trips for treatments that occurred over multiple field trips. We included shark identity (ID) as a random effect to account for pseudo-replication and when the same shark interacted with the deterrent several times within and across trials. We tested for differences in the efficacy of electric deterrents across the three species most responsible for shark bites by sourcing data from previous studies (Table S1) and including species as a fixed factor. 

We determined the most appropriate statistical family for each analysis by examining the distribution of the response variable and visually inspecting model residuals. All models were run for all possible combinations of factors, and compared their probability using Akaike’s information criterion corrected for small sample size (AIC_c_) using the *dredge* function from the MuMIn package (version 1.43.17). Models with Shark ID were run with and without unknown sharks included to test whether the inability to identify all sharks affected our results. In all cases, including the unknown sharks did not decrease the AIC_c_ values of the models, and unknown sharks were therefore excluded from the analyses. We estimated the variance explained by all factors (conditional R^2^; R_c_) and only fixed-factors (marginal; R_m_) using the *r.squaredGLMM* function (package MuMIn version 1.43.17). We estimated marginal means (predicted values) for fixed effects in top ranked models using the *ggpredict* function (package ggeffects version 1.0.1). 

## Results

### Summary of tiger shark results

We ran 70 tiger shark trials for the Freedom+ Surf product (35 Treatment, 35 Control) during daylight hours across 9 days. We removed six trials (5 Treatment, 1 Control) from the behaviour analyses because the video file was corrupted but included them in the analysis comparing the proportion of trials with bites and time for bait to be taken, which we recorded from the vessel. At least 22 tiger sharks interacted with deterrent boards during Freedom+ Surf trials at Norfolk Island (Table S1), and between 1 to 5 sharks were present during each trial. Individuals ranged from 2–4 m total length (TL), with most individuals being 3.2–3.5 m TL. Most sharks were female (13 individuals, 59%), with only two males (9%) and 7 of unidentified sex (32%). One treatment trial was removed from analyses due to lack of approach to the board during the trial, resulting in 35 control and 34 treatment trials. A total of 373 passes were observed (196 during control trials and 177 during treatment trials). Twenty passes (6%) were from individuals that were unidentified.

We did 94 Freedom7 trials (47 Treatment, 47 Control) during daylight hours across five days. We removed nine trials (2 Treatment, 7 Control) from the behaviour analyses because the video file was corrupted but included them in the analysis comparing the proportion of trials with bites and time for bait to be taken, which we recorded from the vessel. Twenty-six tiger sharks were identified at Saunders Reef during Freedom7 trials. Sharks at Saunders Reef were generally smaller than those at Norfolk Island, with individuals estimated between 1.8–3.3 m TL, and most commonly ~ 3 m. Similarly to Norfolk Island, female sharks were dominant (22 vs. 1 male individual, 85%), while 3 individuals could not be sexed (12%). Between 1 and 8 sharks were present during trials. One control trial ended prematurely due to the bait being taken by grey reef sharks (*Carcharhinus amblyrhynchos*)*,* and one treatment was removed due to equipment malfunctioning, leaving 46 control and 46 treatment trials (92 trials total). During Freedom7 trials, 789 passes were observed (218 control, 571 treatment). There were 42 passes (5%) from individuals that were unidentified.

### Probability of bites

**Figure 2:**
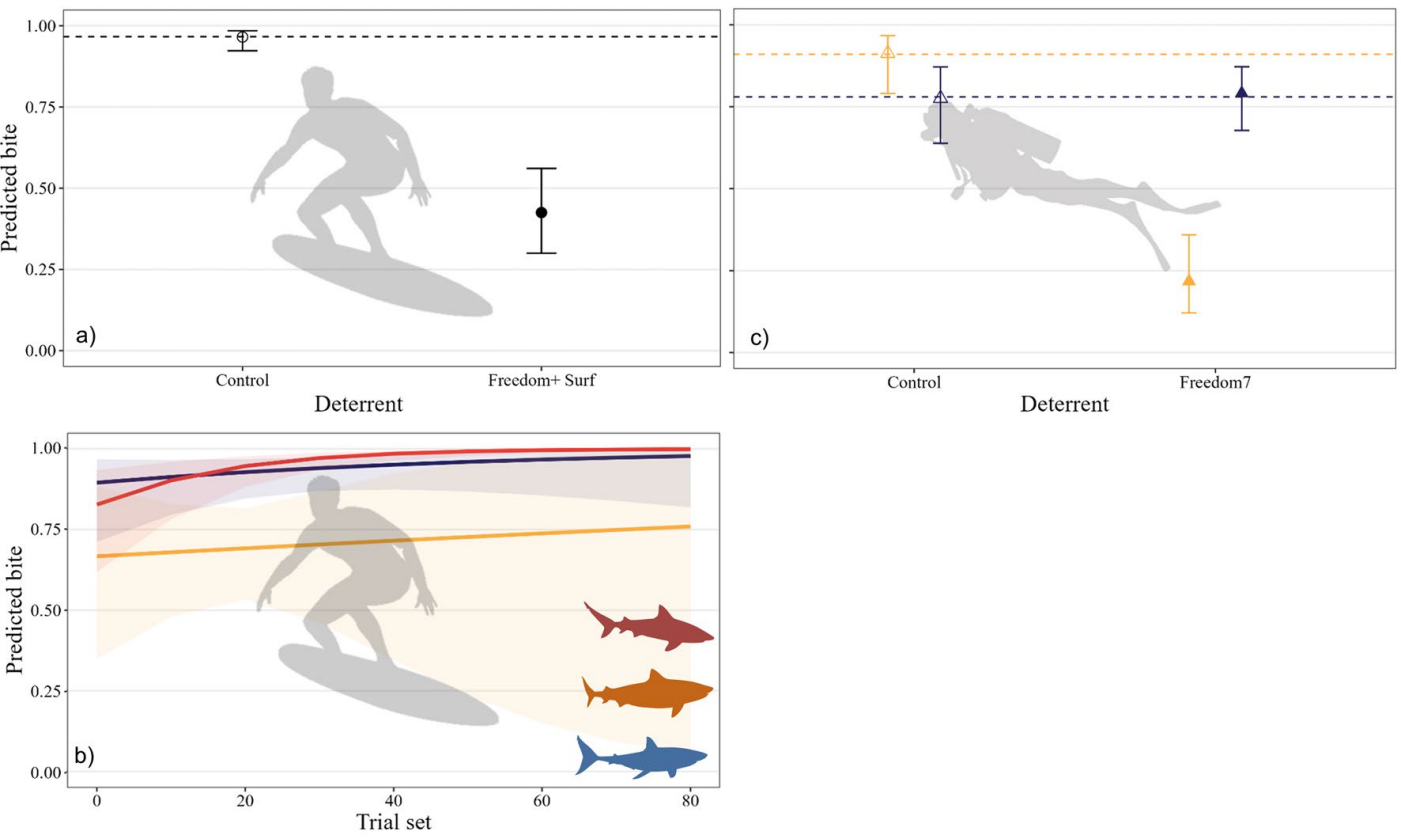
Predicted likelihood (marginal means) of a bite from bull (red), tiger (orange), and white sharks (blue), showing effects of (**a**) Freedom+ Surf deterrent, and interactions between (**b**) species and trial set during Freedom+ Surf trials, and (**c**) species and Freedom7 deterrent. Black symbols indicate no difference between species. Circle symbols indicate Freedom+  Surf and triangles are Freedom7 products. Filled symbols represent active trials, empty symbols are control trials. Horizontal dashed lines indicate mean values during control trials.

The probability of bites from bull, tiger, and white sharks during Freedom+ Surf trials was influenced by deterrent (i.e., whether the deterrent was active or inactive [control]), and the interaction between species and trial set (*w*AIC_*c*_ = 0.69, Table S2a), with 68% of model variance explained from these factors. The probability of bites was reduced by 54% when the Freedom+ Surf was active (Fig. 2a), with the likelihood of a bite occurring decreasing from 0.97 ± 0.43 during control trials to 0.43 ± 0.3. The probability of bites increased with trial number for all three species and regardless of whether the Freedom+ Surf was active (Fig. 2b). White sharks were more likely to bite than tiger and bull sharks at the start of the trial period, gradually increasing in frequency throughout the testing (bite probability increased from 0.9 ± 0.6 to 0.98 ± 1.2; Fig. 2b). Bull sharks were less likely to bite at the start of the testing than white sharks (0.83 ± 0.6, Fig. 2b), but bite frequency increased throughout the trial period to reach the highest probability following 20 trials (up to 0.98 ± 1.2; Fig. 2b). Tiger sharks were the least likely to bite overall, despite a gradual increase in bite probability throughout the trials (0.67 ± 0.78 to 0.76 ± 2; Fig. 2b). Probability of bites during Freedom7 trials was influenced by the interaction between deterrent and species (*w*AIC_*c*_ = 0.89, 33% variance explained, Table S2b). In contrast to Freedom+ Surf trials, there was no change in the probability of bites throughout the trials during testing of the Freedom7 (Table S2b). Bite probability of tiger sharks decreased by 69% when the Freedom7 was active (0.91 of control trial bites vs. 0.22 of treatment; Fig. 2c). The probability of bites from white sharks slightly rose when the Freedom7 was active (1%, 0.78 of control bites vs. 0.79 of treatment; Fig. 2c). Freedom7 has yet to be tested on bull sharks.

### Time for bites to occur

**Figure 3:**
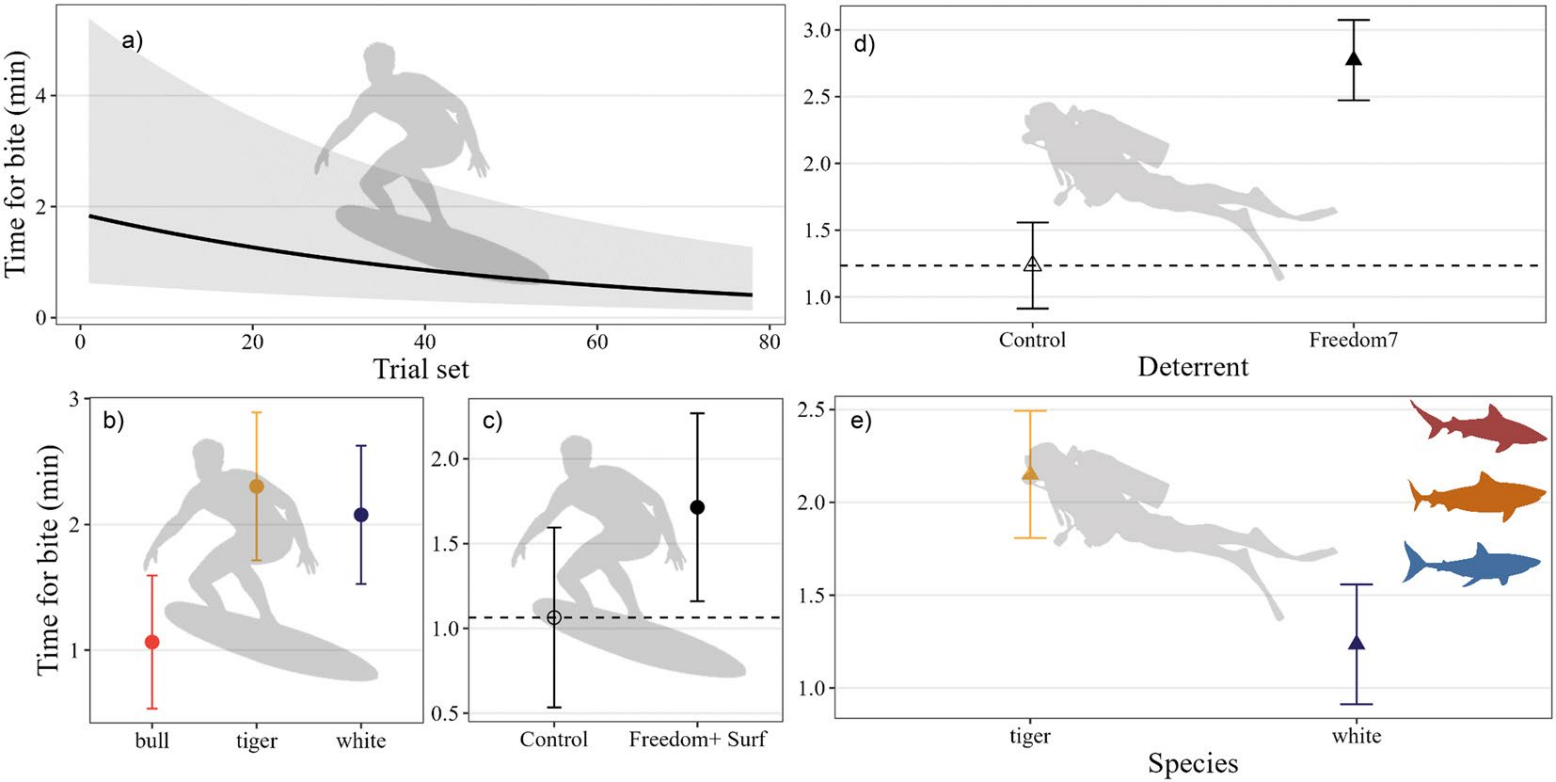
Predicted time (marginal means) of bite on bait or board during (**a**–**c**) Freedom+ Surf and **d**–**e**) Freedom 7 trials. Orange symbols represent tiger sharks, blue is white sharks, and red is bull sharks. Black symbols indicate no difference between species. Horizontal dashed lines indicate mean values during control trials.

The time for a bite to occur during Freedom+ Surf trials was influenced by deterrent, species, trial set, and shark ID (*w*AIC_*c*_ = 0.37, 31% model variance explained, Table S3a), but not the interaction between any of these factors. The predicted time for a bite to occur decreased throughout trial sets (18% model variance explained), from 1.83 ± 0.55 min at the beginning of trials, to 0.41 ± 0.6 min (Fig. 3a). Species had the next highest influence on bite time (16% model variance), with bull sharks biting in nearly half the time (1.1 ± 0.5 min) compared to tiger (2.3 ± 0.6 min) and white sharks (2.1 ± 0.6 min; Fig. 3b). The Freedom+ Surf increased the time for a bite to occur (deterrent = 1% model variance explained), with mean time for a bite increasing by 46% (from 1.06 ± 0.5 during control trials to 1.7 ± 0.6 min when the deterrent was active; Fig. 3c). There was also a high amount of variability in the bite time between individuals, with shark ID contributing 15% of model variance (Table S3a). For the Freedom7, the time for a bite to occur was influenced by deterrent (6% model variance) and species (1% model variance; *w*AIC_*c*_ = 0.31; Table S3b). The Freedom7 deterrent increased the time for a bite to occur by 126%, from 1.24 ± 0.3 min during control trials to 2.8 ± 0.3 min when the Freedom7 was active (Fig. 3d). Time for a bite to occur varied between species, with white sharks biting in a shorter time (1.24 ± 0.3 min) compared to tiger sharks (2.2 ± 0.3 min; Fig. 3e). There was little variation in the time for sharks to bite the bait across individuals, with only 2% of the model variation explained by shark ID. There was no effect of trial (nested within trip number) on the time taken for a bite to occur.

### Number of passes

**Figure 4:**
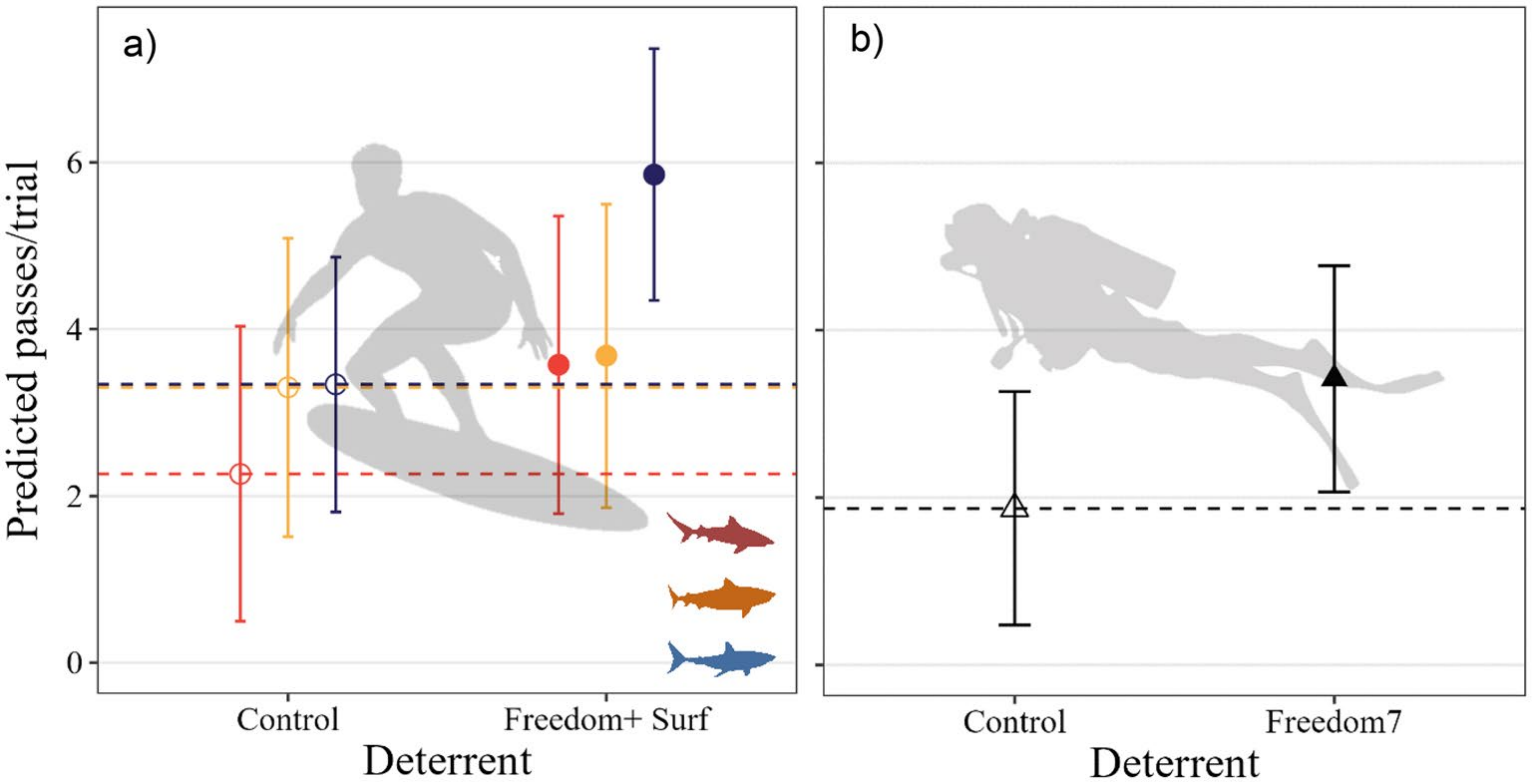
Predicted number of passes (marginal means) from individuals during (**a**) Freedom+ Surf and (**b**) Freedom 7 trials. Orange symbols represent tiger sharks, blue is white sharks, and red is bull sharks. Black symbols indicate no difference between species. Horizontal dashed lines indicate mean values during control trials.

The number of passes during Freedom+ Surf trials was influenced by the interaction between deterrent and species (top-ranked model *w*AIC_c_ = 0.95, 8% model variation). The presence of the active Freedom+ Surf increased the number of passes for all species (Fig. 4a). White sharks had the largest increase in pass frequency, which increased by 54% when the Freedom+ Surf was active (from 3.3 ± 0.8 passes per trial to 5.8 ± 0.8; Fig. 4a). Bull sharks also had an increase in passes when the surf deterrent was active (44% increase, 2.3 ± 0.9–3.6 ± 0.9; Fig. 4a). There was a small increase in the number of passes from tiger sharks during Freedom+ Surf trials (11%), from 3.3 ± 0.9 during control to 3.7 ± 0.9 when the deterrent was absent *vs.* present, respectively (Fig. 4a). Shark identity also influenced the number of passes, with shark ID explaining 13% of the total model variation. Trial set did not affect pass frequency (Table S4a). The number of passes during Freedom7 trials was influenced only by deterrent (5% model variation, Fig. 4b, Table S4b). The number of passes per trial increased by 59% when the deterrent was active, from 1.87 ± 0.7 during control to 3.4 ± 0.7 during treatment (Fig. 4b). There was no effect of species or trial set on number of passes during Freedom7 trials (Table S4b). Individual sharks, however, affected the number of passes, with this factor explaining 3% of the model variation.

### Reactions

**Figure 5:**
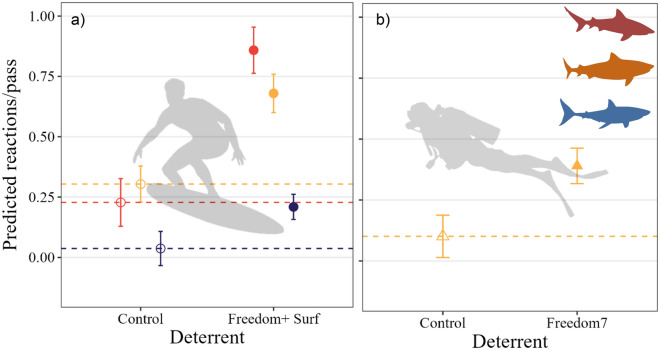
Predicted probability of reactions (marginal means) during passes from tiger (orange), white (blue) and bull sharks during a) Freedom+ Surf, and b) Freedom7 trials. Circles indicate Freedom+ Surf and triangles are Freedom7 products. Filled symbols represent active trials, empty symbols are control trials. Horizontal dashed lines indicate mean values during control trials.

During Freedom+ Surf trials, the probability of reactions during each pass was affected by the interaction between deterrent (i.e., whether the deterrent was active or inactive) and species (*w*AIC_c_ = 0.99, 30% of model variation, Table S5a). Reaction probability increased for all species when the Freedom+ Surf was active (Fig. 5a). Bull sharks had the highest increase in reaction (63% increase, from 0.23 ± 0.05 to 0.86 ± 0.05 reactions per pass), followed by tiger sharks (37%, from 0.3 ± 0.04 to 0.7 ± 0.04), and white sharks (17%, from 0.04 ± 0.04 to 0.2 ± 0.03; Fig. 5a). There was no change in reaction probability throughout trial sets (Table S5a). There was, however, a small effect of individual shark ID on the probability of reactions, with 3% of the variance explained by the random effect (Table S5a). During Freedom7 trials, only deterrent affected the number of reactions per pass (*w*AIC_c_ = 0.99, 7% of model variance, Table S5a). The probability of reactions increased by 28% when the Freedom7 was active (from 0.1 ± 0.04 to 0.39 ± 0.04 reactions per pass, Fig. 5b). There was no temporal effect of trial number (Table S5b). Reaction probability of tiger sharks to the Freedom7 deterrent was also variable between individual sharks, with 7% of the model variance attributed to shark ID. No reaction data was available for white sharks or bull sharks during Freedom7 trials.

### Tiger shark behaviour

**Figure 6:**
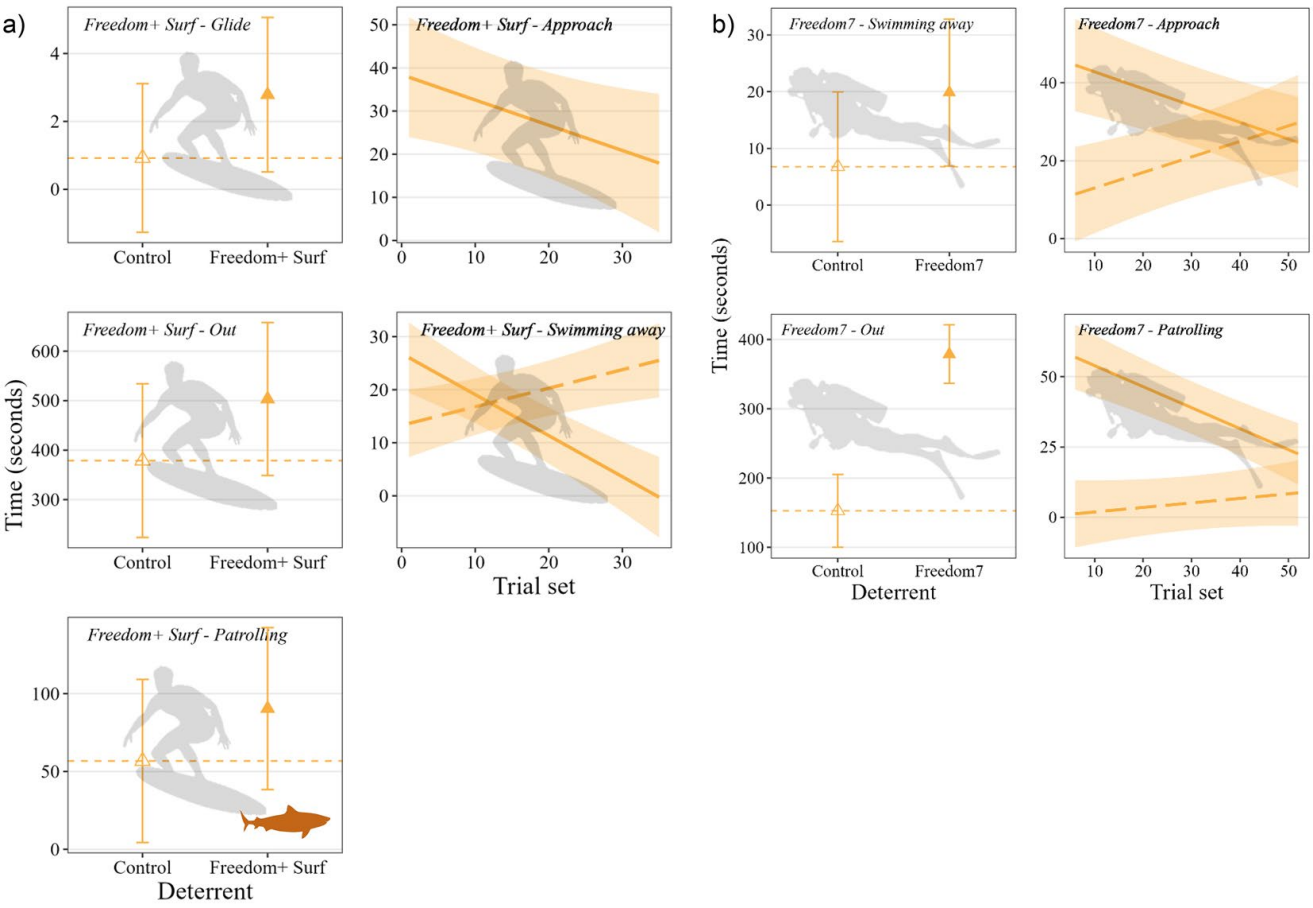
Predicted time (marginal means) that tiger sharks spent across behaviour states during (**a**) Freedom+ Surf trials and (**b**) Freedom7 trials for behaviours included in top-ranked models. Control trials are indicated as empty symbols/dashed lines, and active treatments are filled symbols/solid line.

Behaviour of tiger sharks during Freedom+ Surf trials were coded from 82,507 s (~ 23 h) of footage. This included 21,658 s where the individual shark could not be identified, which was subsequently removed from the analysis, leaving 60,849 s (~ 17 h) of coded behaviours. The Freedom+ Surf product increased the duration that sharks spent gliding (2% of model variance explained by deterrent), were outside field of view (6% model variance), and patrolling (4% model variance; Fig. 6a, Table S6a). The time that tiger sharks spent approaching the bait/boards was not influenced by deterrent but was influenced by trial set (2% of model variation; Table S6a), decreasing throughout the trials (Fig. 6a). Swimming away was influenced by an interaction between deterrent (3% model variation) and trial set (4% model variation), increasing over time during control trials but decreasing throughout the trials when the deterrent was active (Fig. 6a). Duration that sharks spent in each behaviour during Freedom+ Surf trials was also influenced by individual sharks, with 1–14% of each behaviour model variability attributed to shark ID (Table S6a). During Freedom7 trials on tiger sharks, 99,642 s (~ 28 h) of footage was coded. From this, 22,314 s was from unidentified sharks and removed from the analysis, with 77,328 s (~ 21 h) of behaviour data remaining. Duration that sharks spent outside the field of view (*w*AIC_c_ = 0.2, 17% model variance), and swimming away (*w*AIC_c_ = 0.45, 23% model variance) was influenced by deterrent, both increasing when the Freedom7 was active (Fig. 6b, Table S6). Time that sharks spent approaching deterrent setups (*w*AIC_c_ = 0.77) and patrolling (*w*AIC_c_ = 0.64) were both influenced by the interaction between the Freedom7 deterrent and trial set, with the duration spent in these behaviour states increasing throughout the trials for control trials, but decreasing over time during treatment trials (Fig. 6b). Individual identity also influenced the duration that tiger sharks spent in each behaviour state during Freedom7 trials, with 1–5% of the model variance explained by the random effect, shark ID. There was no effect of deterrent, trial set, or shark ID on the time that tiger sharks spent gliding during Freedom7 trials (Table S6b).

## Discussion

This is the first study to test the effects of commercially-available electric deterrents on the behaviour of tiger sharks *Galeocerdo cuvier*, enabling a comparison to the responses of bull and white sharks assessed in previous studies. With the exception of Freedom7 which failed to reduce white shark bites (but see below for likely reasons) and which has not yet been tested on bull sharks, Ocean Guardian electric deterrents decreased the probability of bites from all species tested. Both electric deterrents also increased the time for a bite to occur, number of passes, and probability of reactions across all species. There was, however, intraspecies variability, with some individuals being affected by electric deterrents more than others. Our findings show that both deterrents can reduce the risk of shark bites across bull, tiger, and white sharks, but that the responses to these deterrents vary across species, and between individuals. However, neither the Freedom+ Surf nor Freedom7 deterred sharks completely, and all species were still able to bite the bait when the deterrent was active.

The reduction in the likelihood of being bitten across all three species supports our first hypothesis, that both products reduce the probability of a bite across all species. However, these electric deterrents were not consistently more effective on bull than tiger or white sharks, rejecting our second hypothesis. In some cases, tiger sharks were most affected (e.g., probability of bite with Freedom7), while white sharks had the largest increase in the number of passes (with the Freedom+ Surf) and bull sharks had the largest increase in reaction likelihood (with the Freedom+ Surf). Variation in shark behaviour was also observed during control trials, during which sharks did not always bite the bait even when deterrents were inactive. Such variation in behavioural response suggests some differences among species, but that electric deterrents are overall effective at reducing bites (with the exception of white sharks exposed to Freedom7) across the three species responsible for the most fatal shark bites. This is supported by the interaction between species and deterrent being excluded in the top model in most of our analyses. The lack of reduction in the probability of bites by white sharks exposed to the Freedom7 differs with a previous study showing that the Freedom7 could lead to an 83% reduction in interactions for white sharks (i.e., touch or taking of bait; Ref.^30^). This difference between findings is likely due to the position of the baits in relation to the electrodes of the Freedom7. In^29^, the bait was ~ 2–3 m from the deterrents to reproduce the distance between the deterrent and the head of a user, while^30^ and this study placed the bait next to or between the electrodes (< 0.5 m). The discrepancy between studies therefore shows that while these two products can reduce shark bite risk, it may only do so when the person wearing the device is close to the electrodes and that the position of the electrode is important to ensure protection^30,31^.

In addition to decreasing bites from all species, the ability of the electric deterrents to reduce the risk of shark bites is further supported by the time that it took for sharks to take the bait increasing when either deterrent was active. There was, however, differences in the time for a bite to occur among species, e.g., bull sharks bit nearly twice as fast as tiger and white sharks. Differences in bite times among species may be due to heightened competition during trials or behavioural differences (e.g. boldness). With only one bait accessible, the large number of bull sharks present during the trials (4–8 individuals trial^-1^) may have led to intraspecific competition and to the bait being taken faster than during trials with tiger or white sharks (2–5 and 1–4 individuals trial^−1^, respectively,^66,67^). The number of passes per trial also increased for both deterrents and in all species. The increased amount of time to take the bait and number of passes when the deterrents were active suggests that even though sharks can consume baits, the deterrent can cause sharks to hesitate before biting, offering water users more time to leave the water upon seeing a potentially dangerous shark nearby^68^.

The Freedom+ Surf increased the probability of reactions of bull, tiger, and white sharks. Bull sharks showed the largest increase in reaction (68% increase) compared to tiger sharks (37%) and white sharks (17%). One of the reactions observed was the rapid closure of the shark’s nictitating membrane that can cover the surface of the eye of carcharhiniformes such as bull and tiger sharks^69^. Flickering of the nictitating membrane of tiger sharks has been described around prey items during feeding^70,71^, and close encounters with co-specifics^72^. The structure of this membrane protects the surface of the eye from injury during quick manoeuvres during hunting^73^, but also when opening the jaw^74^. The increase in reactions when deterrents were active is likely linked to a direct effect of the electric pulse on nerves or muscles that control the membrane. White sharks (lamniformes) do not possess a nictitating membrane^75^ and instead roll their eye back into the orbit during predation^76,77^. Reactions tested by^28,29^, i.e., tail flick, muscle spasm, head shake, fast direction change, did not include eye rolls, so were not recorded if they occurred in response to the deterrent. Bull sharks showed the largest increase in reactions, which may indicate that their electroreception is more prone to disruption than tiger and white sharks. Reactions were also observed during control trials for all species, albeit less frequently, showing that sharks may naturally perform these reactions during feeding or the presence of an unfamiliar object. For a more extensive understanding of the influence of shark electroreception influencing deterrent efficiency, a more comprehensive knowledge of pore functionality and response to powerful electric fields is needed.

Studies of shark electrosensory systems have shown that some species become habituated to electric stimuli, which can change the response of individuals exposed to these fields (e.g., electromagnetic fields; Ref.^55,78–80^). As a result, there have been concerns about the potential for learning or habituation to the strong electric fields emitted by deterrents, resulting in a decrease in their effectiveness over time. Previous studies that tested whether behaviour responses to electric deterrents changed throughout the study have had inconsistent findings. Some habituations were observed in bull and white sharks^30,31^, but no temporal effects were detected in other studies^29,54,68^. Here, we found limited evidence of habituation for tiger sharks, with behaviours remaining mostly consistent throughout the trials. When behaviour changed over time, e.g., proportion of bites and time of bite with the Freedom+ Surf, the electric deterrents did not change the response (i.e., the interaction between deterrent and trial set was not included in the models). The only case when habituation to deterrents is supported by our data is for the amount of time in approach behaviour during Freedom7 testing. The other two instances when the interaction between deterrent and trial set was included in our detailed behaviour analysis did not support a habituation effect, e.g., patrolling behaviour decreased over time when the deterrent was off, but remained relatively consistent when the deterrent was on. While habituation has been detected in some studies^30,31^, the conditions under which deterrents are tested expose sharks to electric deterrents far more frequently than would occur in a normal context. For example, habituation in bull sharks was detected when 75 active trials occurred over 18 days^31^, which would not happen in real-world applications where shark-human interactions are far less frequent.

The need for sufficient replicates required a study location where many sharks aggregate and the use of berley to attract them. We therefore acknowledge that testing deterrents using natural prey as an attractant in an area with a large number of sharks presents an extreme situation, which is a different context to that of most swimmers, divers, or surfers. The behavioural response of sharks to deterrents might therefore be dependent on context^29^. The ability of deterrents to reduce shark-bite risk might then be greater than found in the extreme situation of our testing. Much of the variation in our models was explained by shark ID, indicating that behavioural responses were highly variable across individuals. The reason for this variation is unknown, but might arise from a combination of different levels of satiation, motivation, experiences, dominance hierarchies, or personalities (i.e., behaviour syndrome or consistency of response across situations). Evidence of dominance hierarchy around food sources based on size or sex has been described in bull^81^ and white sharks^82,83^, and to a lesser extent in tiger sharks^64,84^. Similar intraspecific variability in white shark behaviour during deterrent trials has been noted [e.g., 28, 53], emphasising the need to ensure that shark deterrents are tested on a sufficient number of individuals to identify and account for such individual variability.

Our novel method to examine shifts in tiger shark behaviour during deterrent trials offers an insight into behaviour shifts which may be missed from standard metrics used to test the effectiveness of shark deterrents previously (e.g., probability and time of bites, distance to bait). Previous deterrent studies have often used stereo-video systems which enable measurements to be taken such as nearest distance to bait or deterrent equipment, and to obtain accurate estimates of individual shark size. However, these designs are limited to only record in the direction that the camera is facing (toward deterrent and bait setups), and are constrained to narrow field of views (typically ~ 127° horizontal, ~ 93° vertical; Ref.^85^). Our approach using a 360° video camera and times-series of behaviour states builds on these previous approaches to obtain a complete 360° view around deterrent setups and detect changes in behaviour that would otherwise be missed from conventional approaches. We showed that tiger sharks spent more time outside the field of view of 360° cameras when electric deterrents were active, suggesting that sharks may leave the immediate surrounding area, which had not been noticed previously when only using front-facing cameras. Gliding behaviour, while occurring infrequently, increased in frequency when the Freedom+ Surf was active. Passive swimming, such as gliding on descent or patrolling, can lead to up to 50% in energy saving compared to active swimming^63,86^. Most predatory epipelagic sharks and fishes commonly glide to regulate efficient prey searching and energy saving through oscillatory movements, and can be an effective foraging strategy^63,87,88^. The increased frequency of this behaviour when deterrents are active may therefore be indicative of an energetic-saving strategy by sharks. Our findings show that a 360°field-of-view can detect subtle changes in shark behaviour, in addition to the standard variables coded during deterrent testing (e.g., likelihood of bites, distance to deterrents, number of pass and approach). However, the benefits of the 360° design varies and depends on the environment where testing occurs. For example, deterrents on bull sharks in this study were tested from a wharf where low water visibility and the wharf hampered the ability to record additional behaviours and reduced the benefit of a 360° field-of-view.

## Conclusions

Bull, tiger, and white sharks are cumulatively responsible for the most bites in Australia, along with the highest proportion of bites that result in fatality^9^. Public sentiment towards mitigation measures is increasingly shifting from traditional lethal measures (e.g., drumlines, beach netting) to non-lethal alternatives such as personal deterrents^21^. Our study shows that Ocean Guardian’s Freedom7 and Freedom+ Surf electric deterrents are effective, non-lethal devices that reduce the risk of bites from bull, tiger, and white shark by 54–69%. Behaviour response also varied among individuals, suggesting that deterrent efficacy is affected by shark motivational state or personalities. Although both electric deterrents can reduce the risk of shark bites for surfers and swimmers/divers, neither product eliminated bites from sharks entirely, and while a combination of mitigation measures is necessary to reduce risk further, electric deterrents are an effective and important part of the toolbox of shark-bite mitigation measures.

### Supplementary Information

Supplementary Information.

## Data Availability

The datasets used and/or analysed during the current study available from the corresponding author on reasonable request.
