# Supplementary material for: Personal electric deterrents can reduce shark bites from the three species responsible for the most fatal interactions
Source: Sci Rep. 2024 Jul 15;14:16307. doi: 10.1038/s41598-024-66679-6 (PMC11251019; doi:10.1038/s41598-024-66679-6)
Supplement: Supplementary file 1 — Supplementary Information. [file 41598_2024_66679_MOESM1_ESM.pdf]

Personal electric deterrents can reduce shark bites from the three species responsible for the most fatal interactions - Supplementary material

Thomas M. Clarke, Adam Barnett, Richard Fitzpatrick, Laura A. Ryan, Nathan S. Hart, Arnault R. G. Gauthier, Tracey B. Scott-Holland, Charlie Huveneers

Table S1. Summary of studies testing the effectiveness of Ocean Guardian Freedom+ Surf and Freedom7 models on tiger, white, and bull sharks, including number of trials, sharks, and passes.

Species	Product	Citation	Trials (control/treatment)	Sharks	Passes
Bull shark *Carcharhinus leucas*	Freedom+ Surf	(Gauthier et al. 2020)	75 / 75	29	455
Tiger shark *Galeocerdo cuvier*	Freedom+ Surf	Current study	35 / 34	22	373
	Freedom7	Current study	47 / 46	26	789
White shark *Carcharodon carcharias*	Freedom+ Surf	(Huveneers et al. 2018)	71 / 83	44	640
	Freedom7	(Huveneers et al. 2013)	57 / 75	18	527

Table S2. Model summaries of the top four models estimating effects of a) Freedom+ Surf, and b) Freedom7 on the likelihood of a bite from bull, tiger, and white sharks. AIC_c: Akaike's information criterion corrected for small sample size; ΔAIC_c: difference in AIC_c between the current and top-ranked model; $wAIC_c$: model probability; R_m: marginal (fixed effects) R^2. All models include shark ID as a random factor (1|ID).

Model	*df*	logLik	AIC_c	ΔAIC_c	$wAIC_c$	R_m
a) Freedom+ Surf						
**Bite ~ deterrent + + species*trialset**	**7**	**-117.00**	**248.4**	**0**	**0.35**	**0.65**
Bite ~ deterrent*trialset species*trialset	8	-116.67	249.8	1.45	0.17	0.69
Bite ~ deterrent + species + trialset	5	-119.84	249.9	1.51	0.17	0.64
Bite ~ deterrent*species + species*trialset	9	-115.99	250.6	2.21	0.08	0.68
b) Freedom7						
**Bite ~ deterrent*species**	4	-98.11	204.4	0	0.53	0.33
Bite ~ deterrent*species + trial:trip	5	-97.54	205.4	0.95	0.33	0.34

Bite ~ deterrent*species + trial:trip + species*trial:trip	6	-97.32	207.0	2.63	0.14	0.34
Bite ~ deterrent + species	3	-111.12	228.4	23.95	0	0.24

Table S3. Model summaries of the top four models estimating effects of a) Freedom+ Surf, and b) Freedom7 on time for a bite to occur from bull, tiger, and white sharks. AIC_c: Akaike's information criterion corrected for small sample size; ΔAIC_c: difference in AIC_c between the current and top-ranked model; $wAIC_c$: model probability; R_m: marginal (fixed effects) R^2. All models include shark ID as a random factor (1|ID).

**Model**	**df**	**logLik**	**AIC_c**	**ΔAIC_c**	**$wAIC_c$**	**R_m**	**R_c**
**a) Freedom+ Surf**							
**$_{log}$Timebite ~ deterrent + species + trialset**	**24**	**-177.12**	**411.6**	**0**	**0.37**	**0.15**	**0.31**
$_{log}$Timebite ~ deterrent + species*trialset	27	-172.82	412.4	0.78	0.25	0.17	0.33
$_{log}$Timebite ~ deterrent + trialset	25	-175.79	414.0	2.41	0.11	0.15	0.31
$_{log}$Timebite ~ trialset + deterrent*species	26	-175.82	415.2	3.63	0.06	0.15	0.31
**b) Freedom7**							
**$_{log}$Timebite ~ deterrent + species**	**10**	**-161.09**	**344.6**	**0.00**	**0.31**	**0.10**	**0.12**
$_{log}$Timebite ~ deterrent + trialset:trip	10	-161.57	346.0	1.46	0.15	0.11	0.13
$_{log}$Timebite ~ deterrent	10	-161.07	346.2	1.62	0.14	0.08	0.1
$_{log}$Timebite ~ deterrent*species	11	-161.03	346.8	2.26	0.10	0.1	0.12

Table S4. Model summaries of the top four models estimating effects of a) Freedom+ Surf, and b) Freedom7 on number of passes from bull, tiger, and white sharks. AIC_c: Akaike's information criterion corrected for small sample size; ΔAIC_c: difference in AIC_c between the current and top-ranked model; $wAIC_c$: model probability; R_m: marginal (fixed effects) R^2. All models include shark ID as a random factor (1|ID).

**Model**	**df**	**logLik**	**AIC_c**	**ΔAIC_c**	**$wAIC_c$**	**R_m**	**R_c**
**a) Freedom+ Surf**							
**passes ~ deterrent*species**	**42**	**-1158.8**	**2411.6**	**0.00**	**0.95**	**0.1**	**0.23**
passes ~ deterrent + species	39	-1166.0	2417.9	6.29	0.04	0.07	0.20
passes ~ deterrent	41	-1164.1	2420.1	8.47	0.01	0.07	0.20
passes ~ species	35	-1186.5	2449.6	37.96	0.00	0.02	0.15
**b) Freedom7**							
**passes ~ deterrent**	**20**	**-1100.9**	**2243.9**	**0.00**	**0.53**	**0.06**	**0.09**
passes ~ deterrent + species	21	-1100.7	2245.6	1.74	0.22	0.06	0.09
passes ~ deterrent +trial:trip	23	-1099.0	2247.2	3.28	0.10	0.06	0.09
passes ~ deterrent*species	22	-1100.5	2247.7	3.82	0.08	0.06	0.09

Table S5. Models estimating effects of deterrents a) Freedom+ Surf, and b) Freedom7 on the frequency of reactions from tiger sharks, bull sharks, and white sharks. AIC_C: Akaike's information criterion corrected for small sample size; ΔAIC_C: difference in AIC_C between the current and top-ranked model; $wAIC_C$: model probability; Rm: marginal (fixed effects) R^2; Rc, conditional (fixed and random effects) R2. All models include shark ID as a random factor (1|ID).

Model	df	logLik	AIC_c	ΔAIC_c	$wAIC_c$	R_m	R_c
**Reactions (Binomial)**							
**a) Freedom+ Surf**							
**reactions ~ deterrent*species**	**8**	**-558.2**	**1132.6**	**0.00**	**0.99**	**0.30**	**0.33**
reactions ~ trialset + deterrent*species	9	-564.4	1146.9	14.34	>0.01	0.30	0.33
reactions ~ deterrent*species + deterrent*trialset	10	-569.8	1159.7	27.17	0.00	0.30	0.33
reactions ~ deterrent + species	6	-577.2	1166.5	33.99	0.00	0.26	0.3
**b) Freedom7**							
**Reactions ~ deterrent**	**4**	**-478.6**	**965.2**	**0.0**	**0.99**	**0.07**	**0.14**
Reactions ~ deterrent + trialset	5	-483.4	977	11.7	>0.01	0.07	0.15
Reactions ~ 1 (Intercept)	3	-503.7	1013.4	48.2	0.00	0.00	0.90
Reactions ~ trialset	4	-508.9	1025.8	60.6	0.00	>0.01	0.10

Table S6. Models estimating effects of deterrents a) Freedom+ Surf, and b) Freedom7 on time spent in behaviour states by tiger sharks. AIC_C: Akaike's information criterion corrected for small sample size; ΔAIC_C: difference in AIC_C between the current and top-ranked model; $wAIC_C$: model probability; Rm: marginal (fixed effects) R^2; Rc, conditional (fixed and random effects) R^2.

Product	Model	df	logLik	AIC_c	ΔAIC_c	$wAIC_c$	R_m	R_c
**a) Freedom+ Surf**	**approach ~ trialset + (1|ID)**	**8**	**-653.4**	**1325.9**	**0.00**	**0.32**	**0.02**	**0.06**
	approach ~ (1|ID)	7	-655.3	1326	0.06	0.31	0.00	0.04
	approach ~ deterrent*trialset + (1|ID)	10	-651.8	1327.4	1.45	0.16	0.04	0.08
	approach ~ deterrent + (1|ID)	8	-655.3	1328.2	2.28	0.1	>0.01	0.04
	**glide ~ deterrent + (1|ID)**	**4**	**-409.2**	**828.4**	**0.00**	**0.21**	**0.02**	**0.02**
	glide ~ (1|ID)	3	-410.5	829	0.55	0.16	0	>0.01
	glide ~ deterrent	3	-411.4	829	0.57	0.15	0.02	0.02
	glide ~ 1 (intercept)	2	-412.8	829.6	1.19	0.11	0.00	0.00
	**out ~ deterrent + (1|ID)**	**11**	**-862.9**	**1752**	**0.00**	**0.5**	**0.06**	**0.11**
	out ~ deterrent + trialset + (1|ID)	12	-862.8	1753.6	1.59	0.22	0.06	0.11
	out ~ deterrent*trialset + (1|ID)	13	-862	1754.6	2.63	0.13	0.07	0.12
	out ~ deterrent	3	-875	1756.1	4.03	0.07	0.01	0.06
	**patrolling ~ deterrent + (1|ID)**	**13**	**-715.9**	**1461.9**	**0.00**	**0.51**	**0.04**	**0.18**
	patrolling ~ deterrent + trialset + (1|ID)	14	-715.5	1463.1	1.25	0.27	0.04	0.18

	patrolling ~ (1|ID)	12	-718.7	1465.4	3.54	0.09	0.00	0.14
	patrolling ~ deterrent*trialset + (1|ID)	15	-715.5	1465.6	3.76	0.08	0.04	0.18
	**swimmingaway ~ det*trialset + (1|ID)**	**11**	**-534.8**	**1094.2**	**0.00**	**0.90**	**0.12**	**0.14**
	swimmingaway ~ deterrent*trialset	4	-544.4	1099.3	5.04	0.07	0.09	0.11
	swimmingaway ~ deterrent + (1|ID)	7	-543.3	1102.5	8.31	0.01	0.03	0.05
	swimmingaway ~ deterrent + trialset + (1|ID)	8	-542.3	1104.1	9.82	>0.01	0.04	0.06
**b) Freedom7**	**approach ~ det*trialset + (1|ID)**	**15**	**-1001.9**	**2036.4**	**0.0**	**0.77**	**0.07**	**0.12**
	approach ~ deterrent + (1|ID)	12	-1005.9	2039.7	3.4	0.15	0.03	0.08
	approach ~ deterrent + trialset + (1|ID)	13	-1005.8	2041.4	5.0	0.06	0.04	0.09
	approach ~ (1|ID)	11	-1010.7	2044.8	8.5	0.01	0.00	0.05
	**glide ~ 1 (Intercept)**	**2**	**-396.0**	**796**	**0.0**	**0.22**	**0.00**	**0.00**
	glide ~ (1|ID)	2	-396.0	796	0.0	0.22	0.00	>0.01
	glide ~ deterrent	3	-395.7	797.6	1.6	0.10	>0.01	>0.01
	glide ~ deterrent + (1|ID)	3	-395.7	797.6	1.6	0.10	>0.01	>0.01
	**out ~ deterrent**	**3**	**-1443.6**	**2893.3**	**0.0**	**0.20**	**0.17**	**0.17**
	out ~ deterrent + (1|ID)	3	-1443.6	2893.3	0.0	0.20	0.15	0.15
	out ~ deterrent*trialset	4	-1441.8	2893.9	0.6	0.15	0.18	0.18
	out ~ deterrent*trialset + (1|ID)	5	-1441.8	2893.9	0.6	0.15	0.16	0.16
	**patrolling ~ det*trialset + (1|ID)**	**12**	**-1028.8**	**2084.1**	**0.0**	**0.64**	**0.19**	**0.19**
	patrolling ~ deterrent*trialset	4	-1038.0	2086.4	2.3	0.20	0.19	0.19
	patrolling ~ deterrent + trialset + (1|ID)	12	-1030.9	2088.2	4.2	0.08	0.17	0.17
	patrolling ~ deterrent + (1|ID)	11	-1032.0	2088.7	4.6	0.06	0.16	0.16
	**swimmingaway ~ deterrent + (1|ID)**	**13**	**-917.1**	**1862.2**	**0.0**	**0.45**	**0.09**	**0.12**
	swimmingaway ~ deterrent*trialset + (1|ID)	14	-915.7	1863	0.8	0.31	0.13	0.13
	swimmingaway ~ deterrent + trialset + (1|ID)	13	-917.0	1863.4	1.2	0.25	0.12	0.12
	swimmingaway ~ deterrent	3	-934.4	1874.9	12.7	>0.01	0.07	0.07

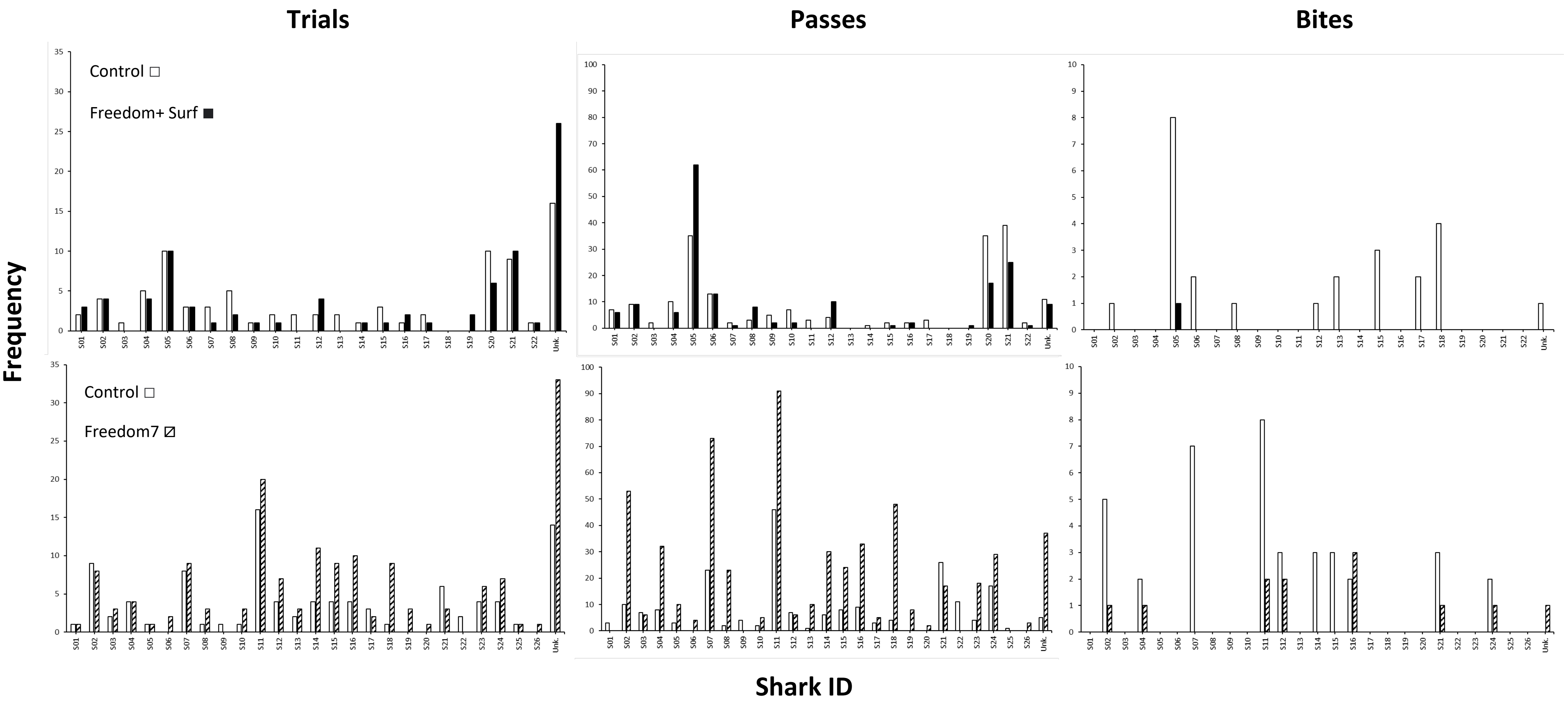

Figure S1. Summary of interactions with tiger sharks during Freedom+ Surf (top) and Freedom7 (bottom) trials
